# Notification that new names of prokaryotes, new combinations and new taxonomic opinions have appeared in volume 75, part 4 of the IJSEM

**DOI:** 10.1099/ijsem.0.006792

**Published:** 2025-07-31

**Authors:** Aharon Oren, Markus Göker

**Affiliations:** 1 The Institute of Life Sciences, The Hebrew University of Jerusalem, The Edmond J. Safra Campus, 9190401 Jerusalem, Israel; 2 Leibniz Institute DSMZ – German Collection of Microorganisms and Cell Cultures, Inhoffenstrasse 7B, 38124 Braunschweig, Germany

This listing of names of prokaryotes published in a previous issue of the *IJSEM* is provided as a service to microbiology to assist in the recognition of new names and new combinations. This procedure was proposed by the Judicial Commission [1]. The names given herein are listed according to the rules of priority (i.e. time and date of publication of the articles on the journal’s platform and the order of valid publication of names in the original articles) [2].

**Table:** 

Order of article publication	Name/authors	Proposed as	Article no.	Page no.
1	*Marinicellulosiphila* Tachioka *et al*. 2025	gen. nov.	006742	8
1	*Marinicellulosiphila megalodicopiae* Tachioka *et al*. 2025	sp. nov.	006742	10
2	*Vallitalea sediminicola* Hirano *et al*. 2025	sp. nov.	006721	7
2	*Vallitalea maricola* Hirano *et al*. 2025	sp. nov.	006721	8
3	*Nocardia arseniciresistens* Wang *et al*. 2025	sp. nov.	006738	6
4	*Amphibiibacter* Costa *et al*. 2025	gen. nov.	006741	9
4	*Amphibiibacter pelophylacis* Costa *et al*. 2025	sp. nov.	006741	11
5	*Sinomonas puerhi* Fu *et al*. 2025	sp. nov.	006752	5
6	*Phocoenobacter skyensis* (Birkbeck *et al*. 2002) Nilsen *et al*. 2025	comb. nov. [basonym: *Pasteurella skyensis* Birkbeck *et al*. 2002]*	006729	7
6	*Phocoenobacter atlanticus* Nilsen *et al*. 2025	sp. nov.	006729	7
6	*Phocoenobacter atlanticus* subsp. *atlanticus* Nilsen *et al*. 2025	subsp. nov.	006729	7
6	*Phocoenobacter atlanticus* subsp. *cyclopteri* Nilsen *et al*. 2025	subsp. nov.	006729	7
7	*Streptomyces machairae* Prole *et al*. 2025	sp. nov.	006736	11
7	*Streptomyces achmelvichensis* Prole *et al*. 2025	sp. nov.	006736	11
7	*Streptomyces pratisoli* Prole *et al*. 2025	sp. nov.	006736	11
7	*Streptomyces caledonius* Prole *et al*. 2025	sp. nov.	006736	11
8	*Inconstantimicrobium mannanitabidum* Ueki *et al*. 2025	sp. nov.	006748	10
9	*Albibacterium profundi* Wang *et al*. 2025	sp. nov.	006754	7
9	*Albibacterium indicum* (He *et al*. 2020) Wang *et al*. 2025	comb. nov. [basonym: *Pedobacter indicus* He *et al*. 2020]	006754	8
10	*Flavobacterium anseongense* Jo *et al*. 2025	sp. nov.	006724	7
10	*Flavobacterium wongokense* Jo *et al*. 2025	sp. nov.	006724	9
11	*Streptomyces citrinus* He *et al*. 2025	sp. nov.	006745	7
12	*Methanobrevibacter intestini* Weinberger *et al*. 2025	sp. nov.	006751	10
13	*Sporomusa carbonis* Böer *et al*. 2025	sp. nov.	006677	11
14	*Nocardioides eburneus* Kang *et al*. 2025	sp. nov.	006743	9
15	*Peterkaempfera podocarpi* Putri *et al*. 2025	sp. nov.	006749	8
16	*Ammonicoccus* Yu *et al*. 2025	gen. nov.	006756	7
16	*Ammonicoccus fulvus* Yu *et al*. 2025	sp. nov.	006756	7
17	*Micropruina sonneratiae* Yi *et al*. 2025	sp. nov.	006759	7
18	*Rodentibacter abscessus* Nakahara *et al*. 2025	sp. nov.	006766	4
19	*Kineothrix sedimenti* Biderre-Petit *et al*. 2025	sp. nov.	006750	9
20	*Pantoea phytostimulans* Campillo-Brocal *et al*. 2025	sp. nov.	006764	8
21	*Virgifigura* Li *et al*. 2025	gen. nov.	006771	5
21	*Virgifigura deserti* Li *et al*. 2025	sp. nov.	006771	5
22	*Pontibacter rufus* Park *et al*. 2025	sp. nov.	006755	9
22	*Pontibacter humidus* Park *et al*. 2025	sp. nov.	006755	10
22	*Pontibacter coccineus* Park *et al*. 2025	sp. nov.	006755	11
2	*Dethiothermospora* Fisher *et al*. 2025	gen. nov.	006760	8
23	*Dethiothermospora halolimnae* Fisher *et al*. 2025	sp. nov.	006760	8
24	*Flavonifractor porci* Niu *et al*. 2025	sp. nov.	006767	9
24	*Flintibacter porci* Niu *et al*. 2025	sp. nov.	006767	9

* The basonym is classified as Risk Group 2

The following taxonomic opinions (i.e. the emendation of circumscriptions or the creation of synonyms) do not affect the status of a name under the International Code of Nomenclature of Prokaryotes. These taxonomic opinions can neither be considered as approved nor as disapproved by the International Committee on Systematics of Prokaryotes.

**Table:** 

Name/authors:	Article no.	Page no.
*Actinoplanes capillaceus* Matsumoto *et al*. 2001 pro synon. *Actinoplanes campanulatus* (Couch 1963) Stackebrandt and Kroppenstedt 1988	006744	5
*Actinoplanes campanulatus* (Couch 1963) Stackebrandt and Kroppenstedt 1988 emend. Mo *et al*. 2025	006744	5
*Albibacterium* García-López *et al*. 2020 emend. Wang *et al*. 2025	006757	7
*Micropruina* Shintani *et al*. 2000 emend. Yi *et al*. 2025	006759	7
*Paraglaciecola agarilytica* (Yong *et al*. 2007) Shivaji and Reddy 2014 pro synon. *Paraglaciecola chathamensis* (Matsuyama *et al*. 2006) Shivaji and Reddy 2014	006758	3
*Paraglaciecola chathamensis* (Matsuyama *et al*. 2006) Shivaji and Reddy 2014 emend. Zou *et al*. 2025	006758	3
*Phocoenobacter* Foster *et al*. 2000 emend. Nilsen *et al*. 2025	006729	5
*Phocoenobacter uteri* Foster *et al*. 2000 emend. Nilsen *et al*. 2025	006729	5
